# BioCause: Annotating and analysing causality in the biomedical domain

**DOI:** 10.1186/1471-2105-14-2

**Published:** 2013-01-16

**Authors:** Claudiu Mihăilă, Tomoko Ohta, Sampo Pyysalo, Sophia Ananiadou

**Affiliations:** 1 The National Centre for Text Mining, School of Computer Science, The University of Manchester, 131 Princess Street, Manchester M1 7DN, UK

## Abstract

**Background:**

Biomedical corpora annotated with event-level information represent an important resource for domain-specific information extraction (IE) systems. However, bio-event annotation alone cannot cater for all the needs of biologists. Unlike work on relation and event extraction, most of which focusses on specific events and named entities, we aim to build a comprehensive resource, covering all statements of causal association present in discourse. Causality lies at the heart of biomedical knowledge, such as diagnosis, pathology or systems biology, and, thus, automatic causality recognition can greatly reduce the human workload by suggesting possible causal connections and aiding in the curation of pathway models. A biomedical text corpus annotated with such relations is, hence, crucial for developing and evaluating biomedical text mining.

**Results:**

We have defined an annotation scheme for enriching biomedical domain corpora with causality relations. This schema has subsequently been used to annotate 851 causal relations to form BioCause, a collection of 19 open-access full-text biomedical journal articles belonging to the subdomain of infectious diseases. These documents have been pre-annotated with named entity and event information in the context of previous shared tasks. We report an inter-annotator agreement rate of over 60% for triggers and of over 80% for arguments using an exact match constraint. These increase significantly using a relaxed match setting. Moreover, we analyse and describe the causality relations in BioCause from various points of view. This information can then be leveraged for the training of automatic causality detection systems.

**Conclusion:**

Augmenting named entity and event annotations with information about causal discourse relations could benefit the development of more sophisticated IE systems. These will further influence the development of multiple tasks, such as enabling textual inference to detect entailments, discovering new facts and providing new hypotheses for experimental work.

## Background

Due to the ever-increasing number of innovations and discoveries in the biomedical domain, the amount of knowledge published daily in the form of research articles is growing exponentially. This has resulted in the need to provide automated, efficient and accurate means of retrieving and extracting user-oriented biomedical knowledge [1-4]. In response to this need, the biomedical text mining community has accelerated research and the development of tools. Text is being enriched via the addition of semantic metadata and thus supports tasks such as analysing molecular pathways [5] and semantic searching [6].

Reviews [7] show that, over the last decade, biomedical text mining has seen significant advancements, ranging from semantically foundational tasks, such as named entity recognition [8], coreference resolution [9,10] and relation [11,12] and event extraction [13-17], to more complex tasks, e.g., automatic summarisation [18,19], question answering [20,21], multimedia [22] and even multi- and cross-lingual information retrieval and extraction [23,24]. The heterogenous tools resulting from this research can also be combined into workflows, using systems such as U-Compare [25] and Argo [26]. Furthermore, there has been much interest recently in studying the intentions expressed in text, also known as meta-knowledge [27,28]. This includes, amongst others, recognising sentences which contain speculation [29-32], negation [31-33] or manner [34]. Other researchers who have looked at biomedical articles noticed significant differences between abstracts and full papers regarding structural, morpho-syntactic and discourse features [35] and event and meta-knowledge aspects [36]. Others define various discourse zones and try to determine automatically to which zone a sentence belongs [37].

One of the most important outcomes of the recent research undertaken into biomedical text mining is the large number of newly created, manually annotated corpora. Examples of such resources are the widely used GENIA corpus [38], GENETAG [39] and other corpora from shared tasks, such as BioNLP ST 2009 and 2011 [16,17]. Although these resources have been designed for their target tasks, they are not necessarily restricted to their respective task and can provide support for other tasks as well. Data reuse is both highly demanded and occurs frequently, as it saves important amounts of human effort, time and money. For instance, the GENIA corpus, which initially contained only named entity annotations, has been extended, partially or fully, by various researchers and groups, to include event annotations and meta-knowledge information.

However, until now, comparatively little work has been carried out on discourse relations in the biomedical domain. The notion of *discourse* can be defined as a coherent sequence of clauses and sentences. These are connected in a logical manner by *discourse relations*, such as causal, temporal and conditional, which characterise how facts in text are related. In turn, these help readers infer deeper, more complex knowledge about the facts mentioned in the discourse. These relations can be either explicit or implicit, depending on how they are expressed in text – using overt *discourse connectives* (also known as *triggers*) or not, respectively.

Statements regarding causal associations have been long studied in general language, mostly as part of more complex tasks, such as question answering [40,41] and textual entailment [42]. Despite this, a single, unified theory of causality has not yet emerged, be it in general or specialised language. There are several pieces of work which characterise how annotators perceive causality and the mechanisms they employ to identify it. For instance, some researchers have showed that causality cannot be identified using intuitive testing techniques in a conscious manner [43]. Therefore, they devise an experiment to select features which allow annotators to coherently identify causality, such as rewording, temporal asymmetry, counterfactuality and various linguistic tests. Other, independent results are similar and show that *necessary and sufficient* conditions are not enough to achieve satisfactory inter-annotator agreement and that paraphrasing is a much more useful method [44].

In biomedical science, causal associations between biological entities, events and processes are central to most claims of interest [45]. Many tasks, such as information extraction, question answering and automatic summarisation, require the extraction of information that spans across several sentences, together with the recognition of relations that exist across sentence boundaries, in order to achieve high levels of performance. Take, for instance, the case in example (1), where the trigger *Therefore* signals a justification between the two sentences: because “a normal response to mild acid pH from PmrB requires both a periplasmic histidine and several glutamic acid residues”, the authors believe that the “regulation of PmrB activity could involve protonation of some amino acids”. 

(1) In the case of PmrB, a normal response to mild acid pH requires not only a periplasmic histidine but also several glutamic acid residues. *Therefore*, regulation of PmrB activity may involve protonation of one or more of these amino acids.

Nevertheless, not all causality relations are as obvious as the previous one, where the trigger is explicit and is usually used to denote causality. In example (2), there is an implicit discourse causal association between the first half of the sentence, “This medium lacked Fe3+ or Al3+, the only known PmrB ligands (Wosten et al., 2000), and contained 10 mM MgCl2”, and the latter half, “which represses expression of PmrA-activated genes”. This is due to the fact that, generally, bacterial gene expression could be affected by specific properties of growth media, such as pH and concentration of metals. Therefore, since the repression of the gene expression was observed in a specific condition of a medium, it is implied that the medium in this condition is the cause of the repression and biologists infer a causal association. 

(2) This medium lacked Fe3+ or Al3+, the only known PmrB ligands (Wosten et al., 2000), and contained 10 mM MgCl2, which represses expression of PmrA-activated genes (Soncini and Groisman, 1996; Kox et al., 2000).

Amongst the large number of corpora that have been developed for biomedical text mining purposes, several include the annotation of statements regarding causal associations, such as BioInfer [46], GENIA [38] and GREC [47]. However, these corpora do not include an exhaustive coverage of causal statements. Furthermore, the granularity of the annotation of such statements is limited in several respects, which are described below. Since such corpus resources underlie most currently existing methods for the automatic analysis of biomedical text, there is an opportunity to advance the state of the art in domain-specific IE and text mining through the improvement of annotation schemata, resources and methods in the area of causal association statements.

The development of tools and resources for the automatic analysis of statements of causality is thus of key importance to information extraction and text mining in domain-specific scientific text. In this paper, we provide an overview of how causality is captured in three types of biomedical research efforts, namely biocuration efforts, pathway models and biomedical corpora. We then describe guidelines for the annotation of statements associated with causal relationships in biomedical texts and present BioCause, a corpus that has been created according to these guidelines. Finally, we analyse the causality annotations and the agreement achieved between the annotators.

### Causality in biocuration efforts

General, non-specific physical causation is of obvious interest in biocuration efforts such as the assignment of Gene Ontology (GO) [48] terms to genes to characterise gene functions [49], in part because detailed molecular-level interactions are rarely known when a phenomenon is first observed. For example, an effect due to P_1_ positively regulating the expression of P_2_ through activation of a transcription factor of P_2_ by catalysing its phosphorylation may be first observed, reported and curated simply as P_1_ having a positive effect on the activity of P_2_. Yet, general terms of causality such as “cause” rarely appear in biomedical domain ontologies or other formalisations of the ways in which entities, processes and events are associated with each other. Instead, such formalisations frequently apply terms such as “regulation”, “stimulation” and “inhibition”. Whilst such terms also carry specific senses in biology, their definitions in domain ontologies and use in biocuration efforts show that, typically, their scope effectively encompasses any general causal association.

The definitions of the Gene Ontology are good examples, due to the wide support of the ontology within the biocuration community, the large number of existing annotations and the adoption of the ontology definitions in prominent domain text annotation efforts. These definitions, included in Table 1, are broader than they may initially appear: they explicitly include indirect physical effects (“control of gene expression”) without limitation on the length of the low-level causal chain and, through enumeration, (“frequency, rate or extent”) effectively exhaust the ways in which a process can be affected by another. Specific cases can further illustrate the breadth of these definitions: GO terms such as regulation of multicellular organism growth are used in curation efforts to capture such findings as that the HDAC3 gene regulates the growth of humans – an indirect causal association across multiple levels of biological organisation that involves very complicated and only partially understood molecular pathways.

**Table 1:** GO regulation definitions

**GO ID**	**GO term**	**GO definition**
GO:0050789	Regulation of a biological process	Any process that *modulates* the frequency, rate extent of a biological process. Biological processes are regulated by many means; examples include the control of gene expression, protein modification or interaction with a protein or substrate molecule.
GO:0048518	Positive regulation of a biological process	Any process that *activates* or *increases* the frequency extent of a biological process. Biological processes are regulated by many means; examples include the control of gene expression, protein modification or interaction with a protein or substrate molecule.
GO:0048519	Negative regulation of a biological process	Any process that *stops*, *prevents* or *reduces* the extent of a biological process. Biological processes are regulated by many means; examples include the control of gene expression, protein modification or interaction with a protein or substrate molecule.

Gene Ontology definitions of regulation, positive regulation and negative regulation of biological processes.

The GO definition of regulation of biological process is thus broadly equivalent to the explicitly comprehensive definition “any process that has any effect on another biological process”. Furthermore, in a neutral biological context, the following pairs of statements are roughly synonymous according to the GO definitions:

“A affects B” → “A regulates B”

“A has a positive effect on B” → “A positively regulates B”

“A has a negative effect on B” → “A negative regulates B”

and the following hold :

“A causes B” ≈ “A positively regulates B”

“A prevents B” ≈ “A negatively regulates B”

One should also consider the exact GO synonyms of positive regulation (up regulation, up-regulation, upregulation of biological process and positive regulation of physiological process) and negative regulation (down regulation, down-regulation, downregulation of biological process and negative regulation of physiological process). Thus, whilst the observation that “causation” is rarely considered in general terms in domain curation, text annotation or IE, most of its scope covered in the many efforts that involve the general concept of regulation is physical causation.

### Causality in pathway models

Pathway model curation is a specific biocuration task of particular interest to systems biology [50]. Pathway curation efforts seek to characterise complex biological systems involving large numbers of entities and their reactions in detail using formal, machine-readable representations. The Systems Biology Markup Language (SBML) standard [51] (http://sbml.org) for pathway representation has been applied to a large number of curation efforts.

In particular, the SBML version used by the CellDesigner software [52] (http://celldesigner.org/) has been adopted by major efforts, such as PANTHER [53] (http://www.pantherdb.org/). As such, the SBML/CellDesigner reaction semantics are of significant interest to domain IE efforts seeking to support automatic pathway curation.

SBML reactions are represented as typed associations of three sets of entities: reactants, products and modifiers. The base reaction types are normally specific biomolecular event/process types, such as binding or phosphorylation, and, thus, are out of scope for the study of general causality. However, SBML also allows the ways in which entities modify reactions to be characterised using specific types, summarised in Table 2, together with related GENIA event types (following [54]). Some of the modification types (e.g., Modulation and Inhibition) are generic and used in practice to annotate general physical causal associations whose detailed molecular mechanisms may not be known.

**Table 2:** SBML/CellDesigner reaction modifications

**SBML/CellDesigner**	**GENIA**
Catalysis	Positive regulation
Physical stimulation	Positive regulation
Modulation	Regulation
Trigger	Positive regulation
Inhibition	Negative regulation

Comparison between SBML/CellDesigner reaction modifications and GENIA event types.

### Causality in biomedical corpora

**Figure 1:**
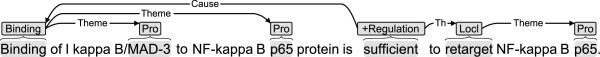
**BioNLP ST GE causality annotation example.** Example annotation from BioNLP shared task GE with annotation for general statement of causality (“is sufficient to”).

**Figure 2:**
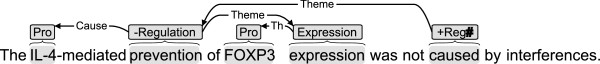
**BioNLP ST GE causality annotation example.** Example annotation from BioNLP shared task GE with annotation for general statements of causality (“prevention” and “caused”).

**Figure 3:**
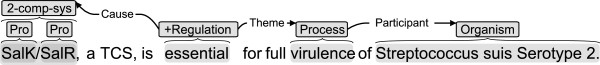
**BioNLP ST ID causality annotation example.** Example annotation from BioNLP shared task Infectious Diseases corpus with annotation for general statements of causality (“is essential for”).

A number of biomedical domain text annotation efforts include statements of general physical causality in their scope. The GENIA event corpus, the most widely adopted manually annotated domain resource for structured information extraction, adopts GO types and annotates statements of general causation using the types Regulation, Positive regulation and Negative regulation[38]. Examples from the GENIA-derived annotation of the BioNLP shared task 2011 GE task corpus are shown in Figures 1 and 2. The GENIA event corpus annotation guidelines have been adapted also to a number of other tasks, such as in the annotation of the BioNLP shared task EPI and ID corpora [55,56]. An example from the ID corpus annotation is given in Figure 3.

**Figure 4:**
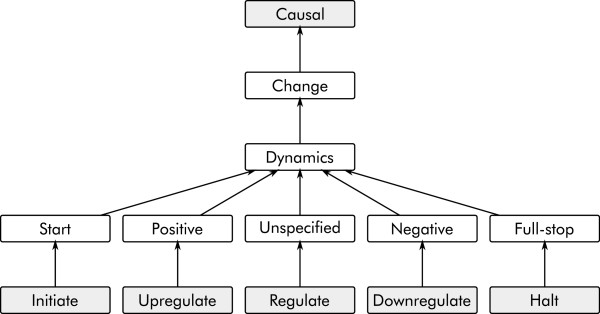
**BioInfer causal ontology excerpt.** Fragment of the BioInfer ontology of causal associations involving change in process dynamics. Arrows correspond to IS-A relationships.

**Figure 5:**
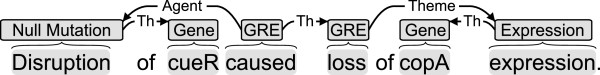
**GREC causality annotation example.** Example annotation from GREC corpus with annotation for general statement of causality (“caused”).

Whilst other domain corpora with similar annotation targets have adopted different ontologies and annotation types, general causality is captured also in the annotation of corpora such as BioInfer [46] and GREC [47]. BioInfer applies an independently developed ontology that incorporates types capturing both the general positive-negative-unspecified distinction involved in GO and GENIA annotation, as well as more detailed subtypes capturing, e.g., the distinction between initiating a process and having a general positive effect on one (Figure 4). In contrast, the GREC corpus opts for an approach where only a small set of specific associations are assigned detailed types, with the majority being generically typed as Gene Regulation Event (GRE). Nevertheless, the scope of this generic type extends to cover also general physical causal associations (Figure 5).

Thus, general physical causality is broadly included in the scope of many domain resources annotated with structured representations for information extraction. However, the scopes of these annotations do exclude a variety of statements potentially involving causal associations. Restrictions include limitation to specific forms of expression such as only verbal and nominalised forms, annotation of explicit statements only and exclusion of statements that only suggest possible causal connections (“A happened after B”). Such limitations imply gaps between the full set of statements of interest and those annotated in domain resources and leave open a number of opportunities for further improvement of resources and tools for the analysis of causality in biomedical text.

Several other more discourse-oriented resources have also been created. The work most similar to ours is the BioDRB corpus [57], which is a collection of 24 open-access full-text biomedical articles selected from GENIA, containing annotations of 16 types of discourse relations, one of which is causality. It was created by adapting the framework of the Penn Discourse TreeBank [58], which annotates the argument structure, semantics and attribution of discourse relations and their arguments. The number of purely causal relations annotated in this corpus is 542. There are another 23 relations which are a mixture between causality and one of either background, temporal, conjunction or reinforcement relations. For machine learning purposes, this dataset is considered relatively small, as it might not capture sufficient contextual diversity to perform well on unseen data. Thus, a detailed comparison and combining this resource with the one described in this article represent an interesting oppurtunity for future work.

## Methods

This section is concerned with the preparatory work required prior to the annotation of the causality corpus. We describe the data that we used and present an overview of the annotation scheme, the annotation tool and an evaluation of inter-annotator agreement.

### Data

It has been shown that there are significant differences between various biomedical sublanguages at the levels of syntax and discourse structure [59], as well as deeper semantics, such as named entity types [60,61]. Therefore, observations made on one sublanguage may not necessarily be valid on another. We thus believe that attempting to train a machine-learning causality detection system on a mixture of subdomains would be detrimental to the learning process. Although we recognise that this choice is associated with high domain specificity, it is preferable to obtain a higher performance in a specific subdomain than a lower performance in a more general domain or a mixture of subdomains. Nevertheless, considering these differences, switching to a different subdomain should be simply a matter of re-training the classifier and re-creating the causality model. Of course, one can extend existing causality models by adding features that have not been encountered before. These would most probably be semantic features, such as a new typology for named entities and events, since these are specific to subdomains.

Furthermore, discourse causality is dependent on the named entities and events present in text. Therefore, in order to isolate the task of recognising causality from that of recognising entities and events, gold standard named entity and event annotations are needed.

Finally, it has been shown that although the information density is highest in abstracts, information coverage is much greater in full texts than in abstracts and thus these may be a better source of biologically relevant data [62,63]. For these reasons, Causality annotation is added on the top of existing event annotations from the BioNLP Shared Task (ST) on Infectious Diseases (ID) [56]. Whilst in other document sets, such as in those used for subdomain analysis [61], entity and event annotations are automatically created by NER and event extraction systems such as NERsuite (http://www-tsujii.is.s.u-tokyo.ac.jp/nersuite/) or EventMine [13], the BioNLP ST ID task has manually created annotations. Furthermore, the BioNLP ST ID corpus has a large size (19 documents) and is comprised of full-text articles.

The existing entity and event annotations have not been be modified in this causality annotation effort even if annotators have spotted mistakes.

### Representation

Conceptually, the annotation involves two basic annotation primitives, spans and relations. Spans represent continuous portions of text with an assigned type, whilst relations are directed, typed, binary associations between two spans. Spans mark both the specific statements in text that play the roles of *Cause* and *Effect* in statements of causality, as well as expressions that explicitly state the existence of a causal relation.

The annotation involves two span types: Argument and Trigger. The former is used to mark statements that are part of a causal relationship, whilst the latter is used to mark phrases that express causal triggers. For instance, in example (3), the text spans “*A occurred*” and “*B happened*” would be marked as Argument, whilst the text span “*Thus*” as Trigger. 

(3) A occurred. *Thus*, B happened.

On the other hand, relations identify connections between the various spans of text. The relation types identify the roles that the spans of text play in the association. The annotation involves three relation types: Effect, Cause and Evidence. Effect always marks the statement that is stated as the result, whilst Cause or Evidence mark the statement that leads to that result. All of these concepts are detailed below.

### Causality

The sense type “Cause” is used when the two arguments of the relation are related causally and are not in a conditional relation. As previously mentioned, this definition is rather vague, so annotators must also use other methods in order to recognise causality. Thus, considering previous research [43,44], they were asked to check for temporal assymetry and counterfactuality, try rewording and other linguistic tests.

Causality annotations are defined with reference to the following two discourse relation subtypes, in a similar manner to the BioDRB corpus. The relation subtype pair *Reason/Result* represents physical causality, whilst the other pair, *Claim/Justification*, represents causality within the discourse, rather than in the physical world it describes. *Reason/Result* holds when the situation described in one of the arguments is the cause of the situation described in the other argument. The other subtype, *Claim/Justification*, holds when the situation described by one of the arguments is the cause, not for the situation described by the other argument, but rather for the truth or validity of the proposition described by the argument.

#### Cause–Reason/Result

**Figure 6:**
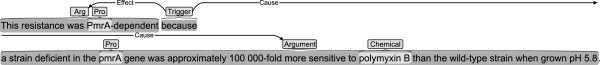
**Explicit trigger reason/result.** Example of Cause–Reason/Result annotation with an explicit trigger.

**Figure 7:**
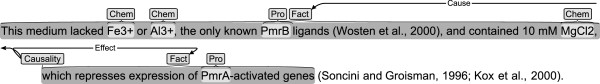
**Implicit trigger reason/result.** Example of Cause–Reason/Result annotation with an implicit trigger.

*Reason/Result* pairs are annotated as centred on a Trigger span, whilst the associated spans are of type Argument, with Cause and Effect representing the direction (Figure 6). The span identifying the causal trigger (Trigger) may be empty, but a non-empty span is marked in all cases where an explicit connective occurs. In cases where there is no explicit connective expressed, the Trigger span is placed in between the two Argument spans with an empty (zero-width) span, as shown in Figure 7.

#### Cause–Claim/Justification

**Figure 8:**
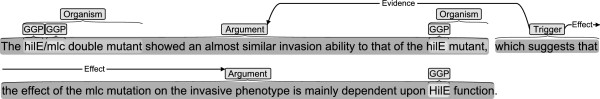
**Explicit trigger claim/justification.** Example of Cause–Claim/Justification annotation with an explicit trigger.

*Claim/Justification* pairs are also annotated as centred on a Trigger span and the associated spans are of type Argument. However, unlike with *Reason/Result* pairs, Evidence and Effect relation types are used to represent the directionality (Figure 8). Similarly to *Reason/Result*, a Trigger span is always marked, using a zero-width span in cases where no explicit trigger appears.

### Scope

All statements of causality falling within the scope of the annotation target should be marked. Consequently, any two possible spans that are not connected by causality annotations (implicitly) represent a “negative” example.

Argument and Trigger annotations should be created only as required for annotating associations between them, e.g., statements that are not part of any annotated association should not be marked. Thus, Argument and Trigger annotations are not exhaustive.

Statements of association other than those annotated as *Causality* are not in the scope of the annotation and are only defined for the reference of the annotators. Consequently, the primary purpose of permitting annotations other than *Causality* is to provide annotators a way to communicate the reason why a specific candidate pair was not marked as *Causality*. This annotation is entirely optional and does not need to be exhaustive. Thus, it has not been included in the final version of the corpus and its consistency is not considered in determining inter-annotator agreement.

All discourse relation types, as defined in BioDRB, are tentatively defined in the annotation tool as relation types. They are represented as relations directly associating Arguments and Triggers do not need to be marked to identify these associations. Some of these relation types, such as *Background* and *Purpose*, could be potential candidates for extending the scope of the *Causality* annotation.

### Annotation software and format

The original event annotation of the BioNLP ID Shared Task corpus was performed using brat[64]. This is a web-based annotation tool aimed at enhancing annotator productivity by simplifying and automating parts of the annotation process. Customising the settings of brat is reasonably straightforward, allowing users to change the information to be annotated and the way it is displayed. Furthermore, brat is freely available under the open-source MIT licence from its homepage (http://brat.nlplab.org). As such, we decided to continue to use this tool for our task of annotating causality relations in text.

**Figure 9:**
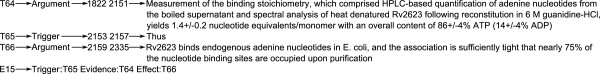
**Annotation format example.** Example of an annotation file as created by BRAT.

The stand-off annotation files are kept separate from the original text files and are connected to them by character offsets. Each span annotation (Trigger and Arguments) has a unique identifier and encodes the start and end offsets of the text span, the type of the span and the actual text span annotated, all separated by tabs. Each causal relation has a unique identifier and stores the identifiers of the trigger and the two arguments, together with their relation subtype. An example of a complete relation annotation is illustrated in Figure 9.

This simple, yet highly efficient format allows for easy processing and full transformation into other formats (e.g., XML), thus increasing the portability between various annotations systems. Furthermore, since this schema is not very specific, it can be reused and easily applied to other datasets, not necessarily belonging to the biomedical domain. Moreover, being represented in an offset stand-off format, the schema can allow the existence of other annotations over the same source text without creating annotation conflicts, such as overlapping in XML. In this case, the text is already annotated with named entity and event information. Other types of annotation are allowed and can be successfully integrated (e.g., part-of-speech and dependency).

### Annotators and training

Although it has been shown that linguists are able to identify certain aspects in biomedical texts reliably, such as negation and speculation [31], they could be overwhelmed in trying to understand the semantics. Identifying which events affect which events, especially when a causal trigger is not explicitly stated, is an extremely difficult task, as it requires vast, domain-specific background knowledge and an almost complete understanding of the topic. Therefore, due to the specificity of the biomedical domain, it is necessary for the annotators to be experts in this field of research. Furthermore, the annotators must have near-native competency in English.

For the purpose of this task, two human experts have been employed to create the annotations in the corpus. One of the annotators is the second author of this article.

Besides the biomedical expertise, the two selected annotators also have extensive experience in annotating text from the biomedical domain for text mining purposes. They have previously participated before in other annotation efforts focussing on creating gold standard corpora of named entities, events and meta-knowledge. The annotators undertook a period of training prior to commencing the annotation task proper. During this time, they were given a small set of documents to practice on. As a result, they became accustomed to both the annotation tool and the guidelines.

Both annotators were given the same subset of articles to annotate, independently of each other. This allowed the detection of annotation errors and disagreements between annotators. They produced annotations in small sets of documents, which were then analysed and in response to which the annotators obtained feedback detailing their errors. Also, the annotators offered feedback regarding the annotation tool and guidelines, in order to increase the speed of the process. This led to noticing potential problems with the guidelines, which were addressed accordingly. The final guidelines were produced after the training period finished and these were used for the actual annotation.

### Evaluating inter-annotator agreement

Due to the complexity of the annotation task and the variety of types of spans and relations, inter-annotator agreement (IAA) cannot be computed using standard means. For instance, the Kappa statistic [65] cannot be used in our case, as this requires classifications to correspond to mutually exclusive and discrete categories. Instead, we have chosen to follow similar cases in selecting F-measure to calculate IAA [47,66].

F-measure is usually used to combine the precision and recall in order to compare the performance of an information retrieval or extraction system against a gold standard. In our case, precision and recall can be computed by considering one set of annotations as the gold standard. The resulting F-score will be the same, regardless of which set is considered gold.

Because of the various angles of annotation, we have split the evaluation methodology into several subtasks of the annotation process. For each subtask, we calculated the inter-annotator agreement in terms of F-score. Initially, we computed the number of identical and overlapping triggers. For these triggers only, we then continued by counting the arguments, using both the exact match criterion and the relaxed match criterion introduced below. This is done separately for the Cause argument and for the Effect argument. 

• Trigger identification – how many causal associations have the same trigger. Two separate values are computed here: 

‒ Exact match – trigger text spans match exactly.

‒ Relaxed match – trigger text spans overlap with each other, but do not necessarily match exactly.

• Argument identification – for agreed triggers, how many have the same arguments. Four separate values are computed here, two for each argument: 

‒ Exact match – argument text spans match exactly.

‒ Relaxed match – argument text spans overlap with each other, but do not necessarily match exactly.

• Relation subtype assignment – for agreed arguments, how often do they have the same relation subtype.

## Results and discussion

In this section, we firstly provide some key statistics regarding the causality annotation produced, together with a discussion of the characteristics of the corpus. Subsequently, we examine the explicit trigger phrases on which the causal relation is centred, followed by an analysis of causality arguments and the distribution of relation subtypes. Finally, we report on the inter-annotator agreement scores on the doubly annotated section of the corpus and investigate the disagreements between the two experts that were found in this part.

### Corpus characteristics and statistics

The causality corpus is freely available under the Creative Commons Attribution Share-Alike Non-Commercial (CC BY-SA-NC) licence from the site of the National Centre for Text Mining (NaCTeM) (http://www.nactem.ac.uk/biocause). The corpus contains a total of 851 causal relation annotations spread over 19 open-access biomedical journal articles regarding infectious diseases.

Table 3 summarises the general statistics of the corpus. Counting the unique explicit trigger types was performed using two settings. On the one hand, we considered the surface expression of the trigger, thus distinguishing between all morphological variants and modifications by adverbs, prepositions or conjunctions. For instance, the triggers *thus* and *and thus* were treated as separate types, as well as *suggest* and *suggests*. However, the case of the triggers was ignored. The tokenisation of triggers and arguments was performed using a naïve regular expression separating tokens when blank spaces, full stops and commas are encountered. As can be seen from the table, there are 381 unique explicit triggers in the corpus. This means that, on average, each trigger is used only 2.10 times.

**Table 3:** General statistics

**Feature**	**Value**
No. of articles	19
No. of causal associations	851
No. of implicit associations	50
No. of unique explicit triggers	381
No. of unique lemmatised explicit triggers	347
Tokens per trigger	3.04
Tokens per Cause arg.	21.22
Tokens per Effect arg.	16.84

On the other hand, all tokens forming triggers were lemmatised prior to counting. This means that both *suggest* and *suggests* are counted for the same trigger type. There are 347 unique lemmatised triggers in the corpus, corresponding to an average usage of 2.30 times per trigger. Both count settings show the diversity of causality-triggering phrases that are used in the biomedical domain.

Furthermore, the causal argument of the relation is, on average, almost 1.32 times longer than the other argument, the effect. This is due to the specificity of the biomedical domain and also the nature of research articles, where usually a causal argument that leads to an effect is complex and is composed of several, concatenated causes. This is exemplified below.

**Figure 10:**
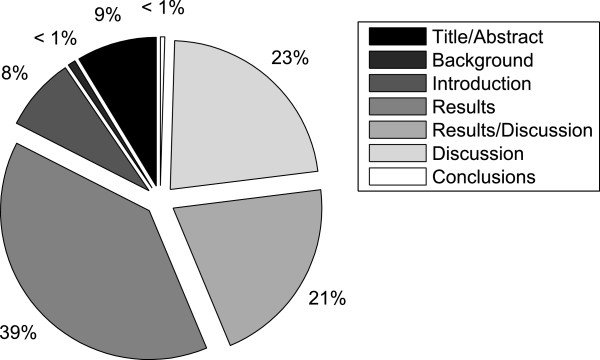
**Causality per discourse zone.** Actual distribution of causal associations in the corpus amongst seven different discourse zones.

We also looked at the distribution of causality relations in the distinct discourse zones that are common in research articles. Figure 10 depicts the percentage of causal relations over six discourse zones, namely *Title and abstract*, *Introduction*, *Background*, *Results*, *Discussion*, *Results and discussion* and *Conclusion*. The zone *Results and discussion* is included because this is how some of the articles have been segmented in their original form.

**Figure 11:**
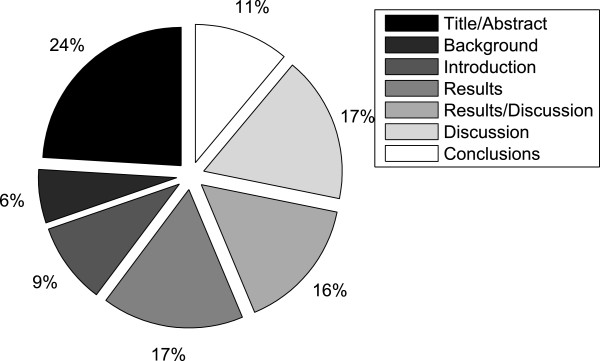
**Causality per discourse zone.** Distribution of causal associations in the corpus amongst seven different discourse zones relative to the number of tokens in each zone.

As expected, most causal relations (over 80%) occur in the *Results*, *Discussion and Results* and *Discussion* section of articles, whereas the *Background* and *Conclusions* section contain a very small number of relations, just over 1%. However, because the discourse zones are very different in size, we also computed the frequency of causal relations relative to the number of tokens present in that respective discourse zone. This distribution is depicted Figure 11. The results change quite dramatically and tend to be more balanced when computed in this manner. The *Title and abstract* section becomes the zone with the highest causal relation density (over 23%), whilst in *Background* and *Conclusion* there are 17%. The *Results*, *Discussion* and *Results and discussion* sections contain 50% of the total number of causal relations.

### Triggers

Table 4 lists the 22 most frequent causality triggers in the corpus, together with their count in the corpus as a whole. These are counted in a surface expression setting. In total, the causality relations that are centred on these 22 triggers constitute more than 30% of the cases of causality in the entire corpus. Similarly, Table 5 contains the 22 most frequent triggers that occur at least five times, counted in a lemmatised setting. These 22 triggers occur 332 times, almost 41.5% of the total number of causality cases. The data in both tables suggest that the majority of relevant causality relations are centred on a relatively small set of phrases and words. Indeed, in the entire corpus, only 22 distinct phrases or words have been used to annotate five or more causal relations, whilst the remaining explicit triggers have a very low frequency of less than five occurrences. As with many other natural language phenomena, this distribution is Zipfian. Almost all of the entries in Tables 4 and 5 correspond to phrases or words which usually denote a causal relation or inference between two spans of text.

**Table 4:** Trigger frequency

**Trigger**	**Count (relative frequency)**
suggesting that	51 (6.04%)
thus	42 (4.98%)
indicating that	34 (4.03%)
therefore	17 (2.01%)
these results suggest that	14 (1.66%)
suggests that	12 (1.42%)
due to	10 (1.18%)
suggesting	10 (1.18%)
indicating	9 (1.06%)
the results indicate that	9 (1.06%)
these results indicate that	9 (1.06%)
suggest that	8 (0.94%)
because	7 (0.83%)
caused	6 (0.71%)
required for	6 (0.71%)
resulting in	6 (0.71%)
which suggests that	6 (0.71%)
and thus	5 (0.58%)
indicates that	5 (0.58%)
suggests	5 (0.58%)
these data indicate that	5 (0.58%)
these observations suggest that	5 (0.58%)

Count and relative frequency for the most frequently occurring triggers using surface-expression forms.

**Table 5:** Trigger frequency

**Trigger**	**Count (relative frequency)**
suggest that	75 (9.36%)
indicate that	45 (5.62%)
thus	42 (5.24%)
suggest	20 (2.50%)
therefore	17 (2.12%)
these result suggest that	15 (1.87%)
indicate	12 (1.50%)
cause	10 (1.25%)
due to	10 (1.25%)
result in	9 (1.12%)
the results indicate that	9 (1.12%)
these result indicate that	9 (1.12%)
because	7 (0.87%)
demonstrate that	7 (0.87%)
which suggest that	7 (0.87%)
lead to	6 (0.75%)
require for	6 (0.75%)
these observation suggest that	6 (0.75%)
and thus	5 (0.62%)
confirm that	5 (0.62%)
our finding indicate that	5 (0.62%)
reveal that	5 (0.62%)

Count and relative frequency for the most frequently occurring triggers using lemmatised forms.

Furthermore, the explicit triggers can be classified into two categories, according to their means of lexicalisation. Firstly, there are triggers which are expressed using subordinating conjunctions or adverbials. These are shown in examples (4) and (5), respectively. There are 37 distinct triggers which belong to this class. 

(4) This acid pH-promoted increase appears to be specific to a subset of PhoP-activated genes that includes pmrD *because* expression of the PhoP-regulated slyA gene and the PhoP-independent corA gene was not affected by the pH of the medium.

(5) Mlc is a global regulator of carbohydrate metabolism and controls several genes involved in sugar utilisation. *Therefore* Mlc also affects the virulence of Salmonella.

The second type is composed of triggers belonging to open-class part-of-speech categories, mainly verbs or nominalised verbs, which are usually modified by conjunctions, prepositions or subordinators. Most of these are of the form subject-predicate, lexicalised as pronoun/noun + verb + adverbial/conjunction/subordinator, where the pronoun/noun is an anaphorical referent to the argument that first appears in the text and the verb shows the relation to the following argument. An instance of this case is shown in example (6), where the verb *suggested* denotes the causal relationship and the subject *This* refers anaphorically to the first sentence. Other patterns also exist, although with a lower frequency, such as prepositional phrases and verb phrases. 

(6) There was residual pbgP expression in the pmrB mutant induced with mild acid pH, which was in contrast to the absence of pbgP transcription in the pmrA mutant. *This suggested that* PmrA could become phosphorylated from another phosphodonor(s) when PmrB is not present.

**Figure 12:**
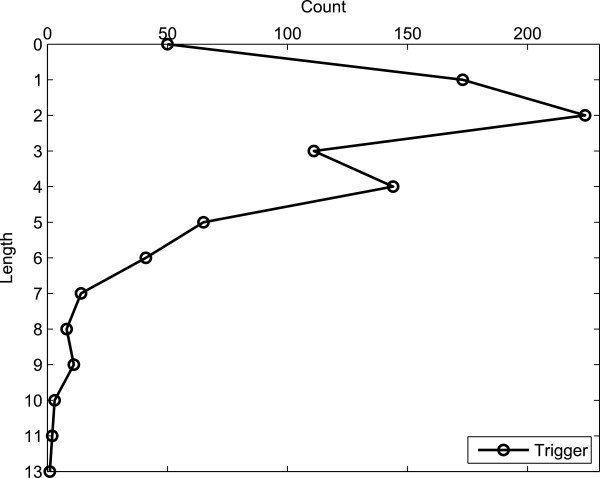
**Trigger length distribution.** Distribution of triggers according to their length in tokens.

We also report, in Figure 12, the distribution of the length of triggers annotated in the corpus, in terms of tokens. As can be seen in the figure, more than 50% of the total number of triggers consist of one or two words, whilst around 25% consist of three or four words. The length of the trigger appears to be inversely proportional to its frequency – the longer the trigger, the more uncommon it is. Again, the distribution has a Zipfian shape.

### Arguments

In order to simplify the explanation we give below and avoid misunderstandings, we will use the following convention: the first argument will always refer to the Cause or Evidence argument of a relation, whereas the second argument will always correspond to the Effect.

**Figure 13:**
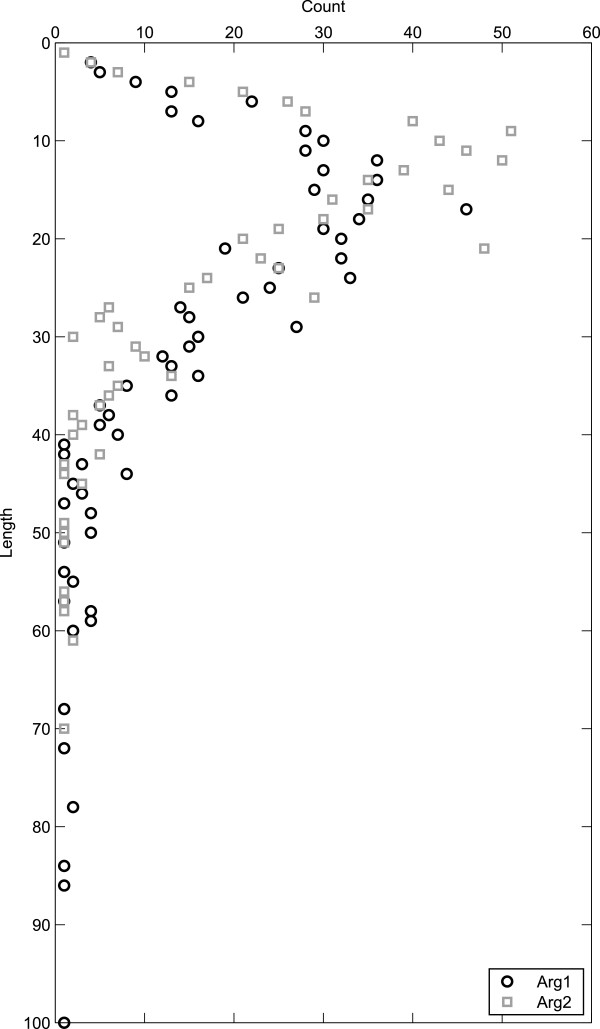
**First and second arguments length distribution.** Distribution of first and second arguments according to their length in tokens. Data points are plotted only where there are instances of arguments of that length.

Figure 13 shows the distribution of the lengths of both the first (black) and the second arguments (grey) in the corpus, in terms of tokens. As previously mentioned, it can be noticed that the first argument is usually longer than the second argument. This is due to the style used in biomedical research articles, in which multiple causal elements are concatenated or explained in order to infer an effect. Take, for instance, the sentences in example (7), where two causal elements (namely “the activation of the hilA transcription” and “that of HilC/D-dependent invFD expression”) are connected by a coordinating conjunction (“and”). Another frequent case is the inclusion of explanations or supplementary information, without which the inference could not be possible. This explains why this information is also included in the argument annotation spans. 

(7) Since HilD activates the transcription of hilA (14), which in turn can activate HilA-dependent invFA expression (10), and directly activates HilC/D-dependent invFD expression, *these results establish that* the mlc mutation exerts a negative effect on SPI1 gene expression, mainly by increasing the level of hilE expression.

The order of the arguments does not vary significantly, with more than 80% occurring in the form of cause-trigger-effect. Table 6 shows the complete distribution of the order of the two arguments relative to the trigger. As can be seen, there are only 24 cases where the trigger appears before or after both arguments. In the case of implicit triggers, we considered them as being placed in between the two arguments.

**Table 6:** Argument order

**Order**	**Count (relative frequency)**
A1-_-A2	30 (3.52%)
A2-_-A1	20 (2.35%)
A1-T-A2	686 (80.61%)
A2-T-A1	91 (10.69%)
A2-A1-T	2 (0.23%)
T-A1-A2	9 (1.06%)
T-A2-A1	13 (1.54%)

Distribution of the order of arguments (A1, A2) relative to the trigger (T). Implicit triggers are marked using an underscore character (‘_’).

**Figure 14:**
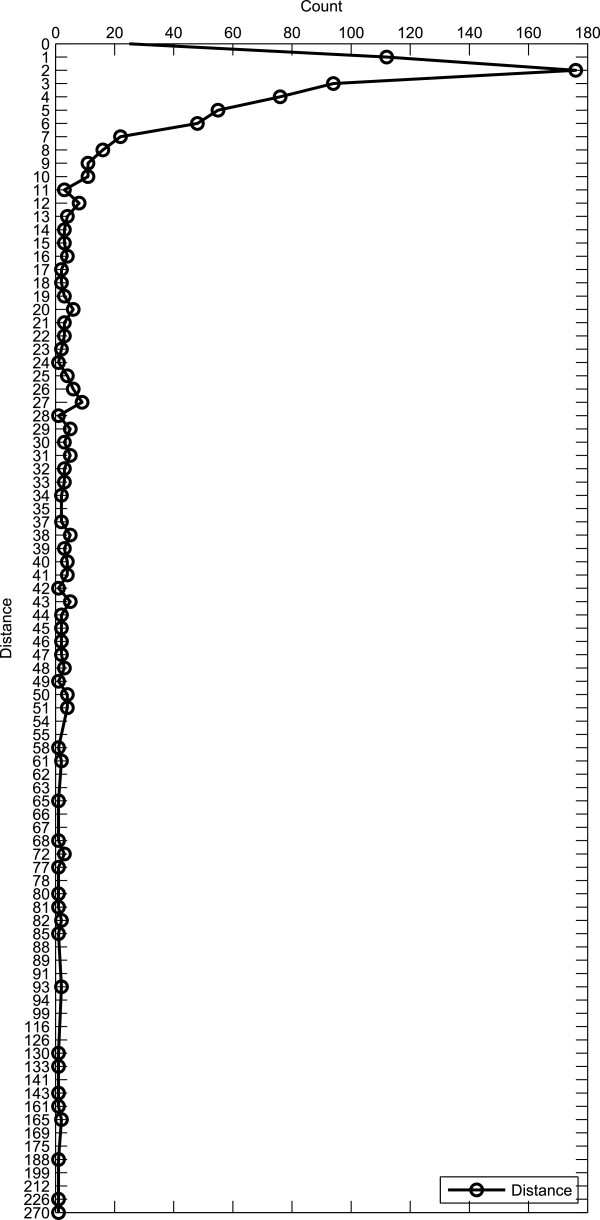
**Distance between arguments distribution.** Distribution of the number of tokens between the first and second arguments.

There are no restrictions on how far the two arguments can be from each other in text. In other words, they may or may not be adjacent. Therefore, we have looked at the distance between the two arguments and show in Figure 14 the frequency of the various distances measured by the number of tokens. The average distance between the two arguments is of 13.5 tokens. It should be noted that this distance also includes the trigger if this is placed in between the two arguments. There are more than one hundred cases where the distance is two or three tokens (116 and 177, respectively). For the distance of four to six tokens, there are between 50 and 100 instances. It can be observed that the graph has a flat, yet long tail. There are almost 200 cases where the distance is greater than or equal to 10 tokens.

### Inter-annotator agreement

In order to ensure the quality and consistency of the causality annotation throughout the corpus, three full articles (approximately 15% of the corpus) were annotated by both human experts. This allowed us to calculate the agreement levels between them. We first present some general agreement statistics on the corpus as a whole, followed by detailed numbers on each subtask. We also analyse the differences in annotation between the two experts.

#### General statistics

Table 7 contains a comparison between the two human expert annotators from various points of view. We included the number of causal associations and their subtypes, the number of implicit and explicit triggers, as well as the average length of the trigger and of the two arguments in tokens.

**Table 7:** General IAA statistics

**Feature**	**First annotator**	**Second annotator**
No. of causal associations	109	125
No. of Evidence arguments	64 (58.72%)	78 (62.40%)
No. of Cause arguments	45 (41.28%)	47 (37.60%)
No. of implicit triggers	13 (11.93%)	18 (14.40%)
No. of explicit triggers	96 (88.07%)	107 (85.60%)
No. of tokens per trigger	2.80	2.87
No. of tokens per Cause arg.	19.55	17.60
No. of tokens per Effect arg.	13.94	13.84

General inter-annotator agreement statistics for the corpus.

As can be observed from the table, there is little difference between the two annotators in terms of the different comparison criteria. The second annotator has identified 16 more causal associations than the first annotator. Nevertheless, the percentage of evidence arguments, cause arguments and implicit triggers remains rather stable over the two sets of annotations. This is also true with respect to the length in tokens of the triggers and the two arguments.

#### Subtask statistics

In order to compute the agreement level in F-score terms, we considered one annotator as the gold standard against which we compare the other annotator. We report in Table 8 the F-scores for the various subtasks. As can be observed, in all the doubly annotated documents, the two annotators agreed, with an exact match criterion, on 60 relations. This gives an F-score of 51.28%, which again proves the difficulty and subjectivity of the task. In the case of relaxed matching, the F-score increases to 65.81%.

**Table 8:** Subtask IAA statistics

	**Feature**	**F-score**
	Exact relation	51.28%
	Relaxed relation	65.81%
	Exact trigger	64.10%
	Relaxed trigger	65.81%
**ET**	Exact Cause arg.	82.67%
	Relaxed Cause arg.	90.67%
	Exact Effect arg.	94.67%
	Relaxed Effect arg.	98.67%
**RT**	Exact Cause arg.	82.52%
	Relaxed Cause arg.	90.91%
	Exact Effect arg.	93.51%
	Relaxed Effect arg.	98.70%

Inter-annotator agreement for relations, triggers and the two arguments in the case of exact-match triggers (ET) and relaxed-match triggers (RT).

The two annotators agreed only on two thirds of the total number of triggers using an exact match criterion. The agreement increases by a small amount when relaxed matching is used. This demonstrates that identifying causal discourse relations is a relatively difficult task, even for experienced human judges.

The agreement on argument spans, nevertheless, is extremely high. This strongly suggests that once the annotators decide to mark a causal relation, finding the arguments is a rather straightforward task to accomplish. The F-score for identifying the Cause argument with an exact match rule is just over 80%, whilst the Effect argument is around 94%. This is due to the difficulty in recognising the exact cause in a causal relation. When the relaxed matching is used, the F-score increases significantly, to 90% for the Cause argument and 98% for the Effect argument.

These agreement values are in line with similar semantic annotation efforts for which F-score has been computed. For instance, in the BioNLP ST ID task, the partial-match inter-annotator agreement for event annotation is approximately 75%. However, the arguments of these events have been already given as gold standard, therefore the task is significantly simpler than the one described in this article. Nevertheless, the best performing system participating in the shared task obtained an F-score of 56%.

After performing the double annotation and computing of the agreement scores, the disagreed cases were discussed between the annotators and the correct annotations were decided upon. Specifically, one of the two annotations was determined to be correct, an alteration was made or the annotation was removed completely. We also computed the agreement of each of the annotator with respect to the resulting gold standard corpus. In an exact-match setting, the F-score of each of the two annotators against the gold standard is 78.26% and 64.68%, respectively. Using a relaxed-match criterion, the F-scores increase to 86.17% and 87.73%, respectively.

### Annotation discrepancies

We also looked at the differences between the two annotators. A number of these differences were simply annotation errors, where the selected spans contained extra characters from surrounding words or missed characters from the words on the boundaries. These have been corrected. The other differences relate to actual disagreements between the two annotators. Similarly to the subtasks on which we computed the agreement scores, the differences can be categorised in those relating to triggers or either of the two arguments.

#### Trigger discrepancies

In the doubly annotated section of the corpus, there are only two cases of overlapping, but not identical, triggers. One of them is given in example (8) below. One annotator considered the span “therein” to be the trigger, whilst the other annotator considered it to be “therein appears to be”. 

(8) Further bioinformatics analysis of the 89K island revealed a distinct two-component signal transduction system (TCSTS) encoded _*Ann*1_[_*Ann*2_[therein ]_*Ann*2_ appears to be ]_*Ann*1_ orthologous to the SalK/SalR system of S. salivarius, a salivaricin regulated TCSTS.

In all other cases, the triggers are either exactly agreed upon or completely distinct. The distinct triggers are not linguistically realised in a different manner than those which were agreed upon. The annotators simply did not agree on considering those cases as suggesting causality.

#### Argument discrepancies

Cases where the two annotators choose overlapping arguments are more frequent than overlapping triggers, but are still insignificant compared to the number of agreed arguments. There are eight cases of overlapping Cause and four of overlapping Effect arguments. Examples for both Cause and Effect are included below, in example (9) and example (10), respectively. 

(9) _*Ann*1_[Results of real-time quantitative RT-PCR also confirmed that, _*Ann*2_[in the complemented strain CDeltasalKR, only partial genes identified as down-regulated in the mutant rebounded to comparative transcript levels of the wild-type strain. ]_*Ann*2_]_*Ann*1_ Those unrecovered genes were probably irrelevant to the bacterial virulence of SS2.

(10) The acid tolerance response of Salmonella results in _*Ann*1_[_*Ann*2_[the synthesis of over 50 acid shock proteins (Bearson et al., 1998) that are likely to function primarily when variations in internal pH occur ]_*Ann*2_, i.e. when Salmonella experiences severe acidic conditions (pH approximately 3). ]_*Ann*1_

In example (9), the Cause arguments chosen by the two annotators overlap. Whilst one annotator considered the entire first sentence as the Cause argument, the other expert did not include the first clause, related to the results. Thus, their argument was annotated as “in the complemented strain CDeltasalKR, only partial genes identified as down-regulated in the mutant rebounded to comparative transcript levels of the wild-type strain”. After discussions, the two annotators agreed to exclude the clause related to the results, as this is not necessary for the correct interpretation of the stated facts.

On the other hand, example (10) shows a case of overlapping Effect arguments. One annotator considered the effect to be “the synthesis of over 50 acid shock proteins (Bearson et al., 1998) that are likely to function primarily when variations in internal pH occur”. The other annotator, however, also included the span of text that further explains and describes the context, “i.e. when Salmonella experiences severe acidic conditions (pH approximately 3)”. The selected argument was the extended version annotated by the first annotator, mainly due to the fact that only the specification of the mentioned condition provides biologists with sufficient detail to correctly understand the biochemical processes that occur in the described situation.

Besides overlapping arguments, there are several cases of completely different arguments. More specifically, there are seven cases of disagreed Cause arguments and only one case of a disagreed Effect argument. As we mentioned above, identifying the Cause argument is a much more difficult task than that of identifying the Effect argument. Since this subtask depends on the background knowledge, expertise and interpretation of each annotator, they might have different biomedical points of view on how events connect to each other causally.

In example (11), we provide one case in which the two annotators select different text spans for the Cause argument of a causal relation. 

(11) _*Ann*1_[In the animal model, attenuation of virulence has been noted for Salmonella strains that carry mutations in the pts, crr, cya or crp genes, which encode the general energy-coupling enzymes of the PTS, enzyme IIAGlc of the PTS, adenylate cyclase and cyclic AMP receptor protein, respectively. ]_*Ann*1__*Ann*2_[Mlc is a global regulator of carbohydrate metabolism and controls several genes involved in sugar utilization. ]_*Ann*2_ Therefore, it seemed possible that Mlc also affects the virulence of Salmonella.

This is due to the fact that Mlc is closely related functionally to the mentioned list of genes (pts, crr, cya and crp). On the one hand, the first sentence provides a more detailed explanation of the cause without mentioning Mlc, together with the observation of the attenuation of virulence. On the other hand, the second sentence mentions Mlc and the genes in general, but it is not linked to the virulence of Salmonella. Thus, the final decision in this case has the first sentence as the cause, since it includes the virulence of Salmonella and the genes that produce it.

### Comparison to the BioDRB

As we have mentioned earlier, the BioDRB contains 542 purely causal relations, as well as 23 relations which are a mixture of causality and other discourse relations. Since the BioDRB and BioCause have similar sizes, we performed a comparison with respect to some of the previous characteristics. The results are included in Table 9. As can be seen, the BioDRB corpus contains a greater number of implicit relations than BioCause. Furthermore, whilst the explicit trigger length is shorter, causal relations in the BioDRB have generally longer cause and effect arguments. The major difference in the order of arguments consists in the lack of the A1-A2-T pattern in BioCause and the lack of the T-A2-A1 pattern in the BioDRB.

**Table 9:** Comparison between BioDRB and BioCause

**Feature**	**BioCause**	**BioDRB**
No. of causal associations	851	565
No. of implicit triggers	50 (11.93%)	98 (17.34%)
No. of explicit triggers	801 (88.07%)	467 (82.65%)
No. of tokens per trigger	3.04	2.46
No. of tokens per Cause arg.	21.22	31.24
No. of tokens per Effect arg.	16.84	20.56
A1-_-A2	30 (3.52%)	78 (13.80%)
A2-_-A1	20 (2.35%)	20 (3.53%)
A1-T-A2	686 (80.61%)	192 (33.98%)
A1-A2-T	0 (0%)	81 (14.33%)
A2-T-A1	91 (10.69%)	135 (23.89%)
A2-A1-T	2 (0.23%)	10 (1.76%)
T-A1-A2	9 (1.06%)	49 (8.67%)
T-A2-A1	13 (1.54%)	0 (0%)

Comparison between BioDRB and BioCause with respect to various measures.

**Figure 15:**
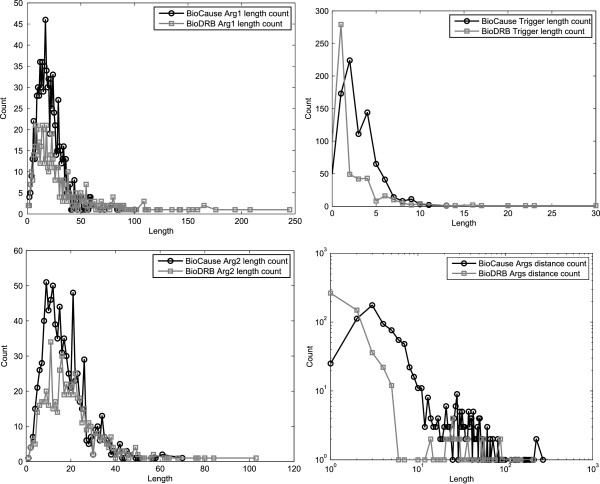
**Comparison between BioCause and BioDRB.** Comparison of the distributions of trigger lengths, first argument lengths, second argument lengths and distance between arguments between the BioCause and BioDRB corpora. The distance between arguments is given using a logarithmic scale in order to provide a better view.

With regard to the distributions of lengths and distances, these are roughly similar in shape when plotted against each other. Figure 15 contains the distributions of trigger lengths, first argument lengths, second argument lengths and distance between arguments between the BioCause and BioDRB corpora. The distribution of the distance between arguments is given using a logarithmic scale in order to provide a better view of the graph for small values. It can be noticed that the first three figures are consistent with the data in Table 9: in BioCause, the triggers are slightly longer, whilst the arguments are slightly shorter.

Considering these similarities and differences, we consider the BioCause and BioDRB corpora as complementing each other. Thus, a future combination of these two resources could prove useful for training a machine learning system capable of recognising causality.

## Conclusions

We have designed and described an annotation scheme for biomedical causality. This scheme captures relevant information regarding causality as it is expressed in biomedical scientific articles, which is of key importance in many text mining tasks undertaken by biologists and biochemists. The scheme is designed to be portable, in order to allow integration with the various different schemes for named entity and event annotation that are currently in existence. Furthermore, the scheme is reusable and extensible, making it possible to apply it to different datasets and to extend it if necessary.

Moreover, we have produced BioCause, a gold standard corpus in which documents from existing bio-event corpora have been manually annotated according to our causality annotation scheme. The annotation was performed by two biomedical experts with extensive experience in producing resources for text mining purposes. We reported a high inter-annotator agreement rate, using both exact match and relaxed match evaluation criteria.

Finally, we have conducted an analysis of the nature of causality as it is expressed in biomedical research articles by examining the annotated corpus. We have described the characteristics of causal triggers and their arguments, looking at distributions of length, frequency and distance.

This corpus will serve as a useful resource for the development of automatic causality recognition systems in the biomedical domain.

## Competing interests

The authors declare that they have no competing interests.

## Authors’ contributions

All authors contributed to the production of the manuscript. SA supervised all steps of the work. CM, TO and SP conceived and designed the annotation scheme and produced the annotation guidelines. CM and TO trained the independent annotator and supervised their annotation and TO performed their own annotation. CM carried out the analysis of the corpus. All authors read and approved the final manuscript.
